# A semi-supervised segmentation method for microscopic hyperspectral pathological images based on multi-consistency learning

**DOI:** 10.3389/fonc.2024.1396887

**Published:** 2024-06-19

**Authors:** Jinghui Fang

**Affiliations:** College of Information Science and Engineering, Hohai University, Nanjing, China

**Keywords:** microscopic hyperspectral images, semi-supervised learning, medical image segmentation, mutual consistency, pseudo-labels

## Abstract

Pathological images are considered the gold standard for clinical diagnosis and cancer grading. Automatic segmentation of pathological images is a fundamental and crucial step in constructing powerful computer-aided diagnostic systems. Medical microscopic hyperspectral pathological images can provide additional spectral information, further distinguishing different chemical components of biological tissues, offering new insights for accurate segmentation of pathological images. However, hyperspectral pathological images have higher resolution and larger area, and their annotation requires more time and clinical experience. The lack of precise annotations limits the progress of research in pathological image segmentation. In this paper, we propose a novel semi-supervised segmentation method for microscopic hyperspectral pathological images based on multi-consistency learning (MCL-Net), which combines consistency regularization methods with pseudo-labeling techniques. The MCL-Net architecture employs a shared encoder and multiple independent decoders. We introduce a Soft-Hard pseudo-label generation strategy in MCL-Net to generate pseudo-labels that are closer to real labels for pathological images. Furthermore, we propose a multi-consistency learning strategy, treating pseudo-labels generated by the Soft-Hard process as real labels, by promoting consistency between predictions of different decoders, enabling the model to learn more sample features. Extensive experiments in this paper demonstrate the effectiveness of the proposed method, providing new insights for the segmentation of microscopic hyperspectral tissue pathology images.

## 1 Introduction

Pathological images are considered the gold standard for clinical diagnosis and cancer grading (1). Automatic segmentation of pathological images is a fundamental and crucial step in constructing powerful computer-aided diagnostic systems. Quantitative analysis of the morphological properties of organs and tissues based on segmentation results provides valuable evidence for clinical diagnosis. Existing pathological image segmentation methods (2–4) typically utilize RGB datasets. However, common RGB images can only provide spatial information for cancer diagnosis. The similarity in biological tissue morphology affects the accuracy of diagnostic results.

With the advancement of imaging systems, medical microscopic hyperspectral images have been employed in various tumor recognition applications (5–7). The DMCA method proposed in (5) integrates the classifier for prediction into the extraction of deep features from MedHSIs. This integration ensures compatibility between the extracted features and the classifier, facilitating tumor diagnosis. In (6), Ravì et al. introduced a novel manifold embedding framework called FR-t-SNE. Using this framework, the outputs generated from hyperspectral imaging can be utilized as inputs for semantic segmentation classifiers of brain tissue *in vivo*. The proposed method aims to delineate tumor boundaries, preserve healthy brain tissue, and facilitate complete removal of malignant cells. Muniz et al. proposed, in (7), a method utilizing hyperspectral imaging and micro-FTIR spectroscopy to represent biological tissues based on their spectral characteristics. Subsequently, a deep learning-based classification approach was established to aid experts in distinguishing tissues affected by cancer or inflammation from healthy tissues. Microscopic hyperspectral imaging applied in medical image analysis relies on the following two fundamental principles: i) tissues with similar biochemical compositions are likely to exhibit similar spectra; and ii) variations in spectra can be quantified to delineate different tissues (8). Compared to conventional imaging modalities, medical microscopic hyperspectral pathological images offer additional spectral information, enabling further differentiation of various chemical constituents within biological tissues. Nevertheless, hyperspectral pathological images have higher resolution and larger area, and their annotation requires more time and clinical experience. Therefore, the lack of precise annotations limits the progress of research in pathological image segmentation.

Semi-supervised learning is a method used to address the issue of limited labeled data. This approach typically involves joint training with a small amount of labeled data and a large amount of unlabeled data. The core of this method lies in effectively extracting useful information from both labeled and unlabeled data to achieve relatively stable segmentation results. To achieve this goal, many semi-supervised algorithms have been applied in this field. Common existing semi-supervised segmentation methods can be categorized into pseudo-labeling and consistency regularization methods (9). Firstly, pseudo-labeling is an intuitive approach where a model trained on labeled data is used to predict pseudo-labels for unlabeled data. These new pseudo-labeled data are then combined with the original labeled set to further refine the model. However, the effectiveness of this method is constrained by the varying quality of the predicted pseudo-labels (9). Consistency regularization methods are based on the smoothness assumption (10). They explore an unsupervised way to leverage unlabeled data. This method (11–13) typically applies slight perturbations to the input data or the model, and learns from unlabeled data by ensuring consistency in model output under different perturbations. Many methods employ a single image to enforce consistency in their perturbations (14), which may lead to inaccurate segmentation results due to a lack of context information across volumes, thus limiting the effectiveness of consistency regularization.

**Figure 1:**
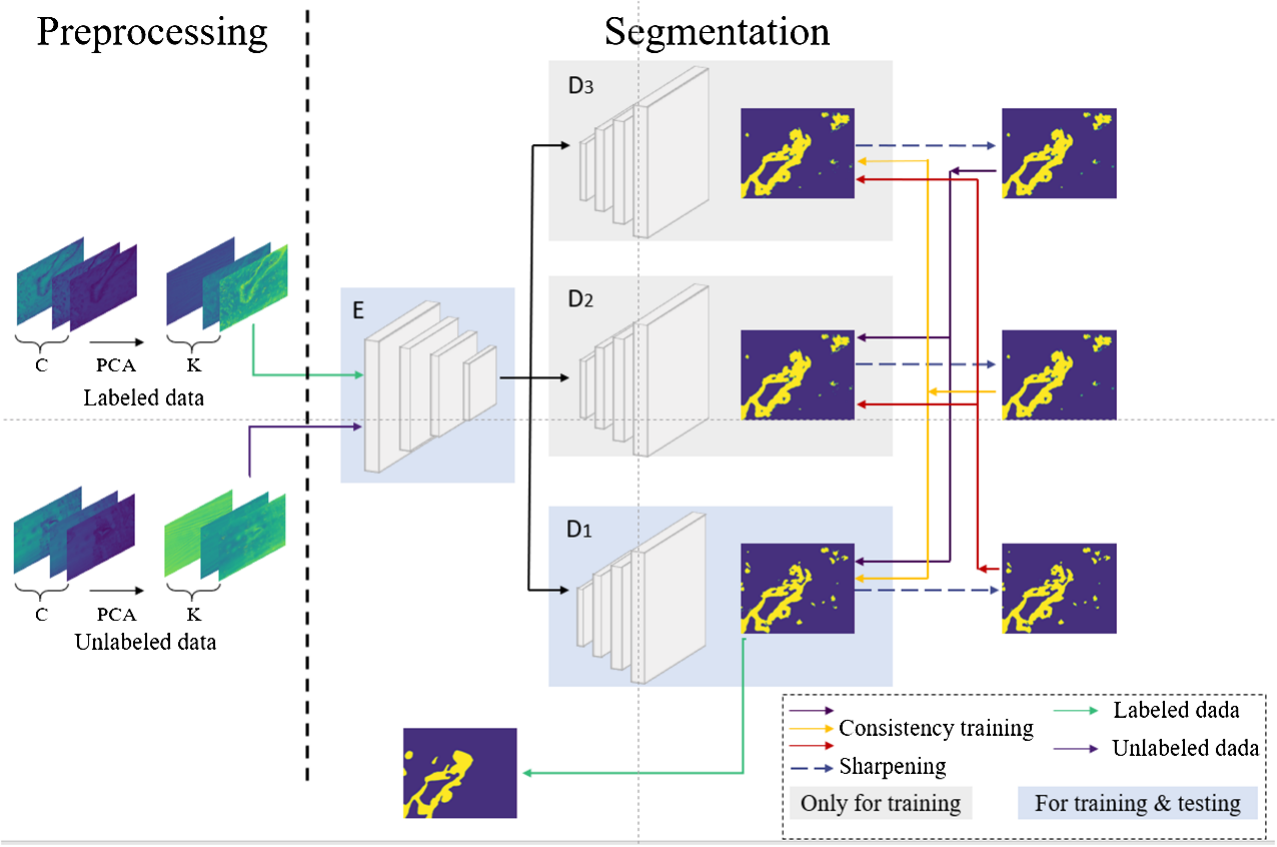
The MCL-Net model proposed in this paper.

Given the aforementioned issues with consistency regularization and pseudo-labeling methods, this paper introduces a semi-supervised segmentation method for microscopic hyperspectral pathological images based on *multi-consistency learning (MCL-Net)*, which combines both methods. The architecture of the model is illustrated in **Figure 1**. The model employs a shared encoder and multiple decoders, where the output of the encoder undergoes different perturbations before being fed into distinct decoders. Subsequently, we employ a novel pseudo-label generation method called Soft-Hard to transform the outputs of different decoders into pseudo-labels. Using these generated pseudo-labels as a basis, we devise a novel multi-consistency training approach, wherein soft pseudo-labels obtained from each decoder are treated as genuine labels for the other decoders and subjected to consistency constraints. Through this approach, we minimize discrepancies in output across multiple decoders during model training, thereby obtaining a more comprehensive feature representation. In summary, this paper makes the following four contributions:

1) A semi-supervised segmentation method for microscopic hyperspectral pathology images based on multi-consistency learning is proposed in this study. This method combines pseudo-labeling and consistency regularization techniques.
2) A multi-consistency learning approach that effectively integrates features extracted by different models is introduced in this research.
3) A novel pseudo-label generation method, Soft-Hard, which generates pseudo-labels that are closer to real labels, has been devised.
4) Extensive experiments demonstrate that our method outperforms five other state-of-the-art methods, providing new insights for the segmentation of microscopic hyperspectral pathology images.

Remaining sections of the paper are organized as follows: Section 2 provides a review of pathological image segmentation and semi-supervised segmentation methods for medical images. Section 3 introduces the MCL-Net method proposed in this paper. Section 4 outlines the experimental setup. Section 5 is dedicated to result analysis. Finally, Section 6 concludes and summarizes the paper.

## 2 Related work

### 2.1 Pathological image segmentation

Pathological images serve as the gold standard for cancer detection, with various segmentation methods being employed in different types of cancer detection. Musulin et al. (15) proposed a two-stage image segmentation method utilizing DeepLabv3+ as the backbone model for predicting oral squamous cell carcinoma in head and neck cancer. Zidan et al. (16) designed a Transformer-based approach constructing a Swin Transformer encoder block to mimic the global context of tumor-related regions in colorectal cancer. Additionally, a cascaded upsampler was devised to utilize supervised multiscale features from the encoder to assist in detecting tumor boundary regions. Jayachandran et al. (17) introduced a novel deep learning framework based on an encoder-decoder structure effectively incorporating attention mechanisms for segmenting osteosarcoma from histological images. Huang et al. (18) proposed an end-to-end ViT-AMCNet, possessing interpretable throat tumor grading capabilities and good interpretability. This model not only ensured good feature representation capabilities of ViT and AMC blocks but also enhanced the redundancy removal ability of the model fusion algorithm. In (19), Rashmi et al. proposed an unsupervised method for segmenting cell nuclei from breast tissue pathology images. A method for selecting template images for color normalization was introduced. An experiment determining a new color channel combination was conducted, which could distinguish cell nuclei from background regions. Furthermore, this work introduced an improved C-V model capable of effectively segmenting nuclei using multi-channel color information. To fully exploit the spectral characteristics of three-dimensional hyperspectral data, Wang et al. (8) applied deep convolutional networks for melanoma segmentation on hyperspectral pathological images. They introduced a 3D fully convolutional network named Hyper-net for segmenting melanoma from hyperspectral pathological images. Zhang et al. (20) proposed a two-stage segmentation method for OSCC tumors with lymph node metastasis. In the learning stage, this method is employed for coarse segmentation of cancer cell nuclei. In the decision stage, the pathologist’s prior knowledge is utilized to make lesion decisions based on the coarse segmentation mask of cancer nuclei, resulting in refined segmentation results. Gao et al. (21) proposed a semi-supervised segmentation method for microscopic hyperspectral pathological images based on shape priors and contrastive learning. They utilized shape priors and image-level contrastive learning to learn features from unlabeled data, enhancing semi-supervised segmentation performance and mitigating limitations posed by limited annotated data. Despite significant progress in tissue pathology image segmentation for cancer prediction, research on hyperspectral pathological images remains limited.

### 2.2 Consistency regularization

Consistency regularization refers to the similarity of predictions generated by a model under the same input data or model with added random noise. It is a crucial component of temporal ensemble techniques (22). Mean Teacher (23) is a classical temporal ensemble technique where both the student and teacher models adopt the same network structure. Through exponential moving average (EMA), the student network’s output across different training iterations becomes similar to that of the teacher network. Various models based on temporal ensemble techniques have been developed based on Mean Teacher. In (24), Shu et al. proposed a novel cross-pollination learning and feature migration mechanism allowing the teacher model to provide higher confidence outputs for student model learning. This method cross-pollinates unlabeled samples to enhance the segmentation network’s generalization ability. It also introduces new cross-gradient monitors to reduce consistency failures caused by semantic gaps between teacher and student models. The average teacher model is enhanced into a novel Fuzzy Consistency Average Teacher (AC-MT) model, where Xu et al. (25) added a series of comprehensive plug-and-play strategies for fuzzy (informative) target selection based on Mean Teacher. This model stabilizes disturbances in regions, enabling more useful representations to be learned from unlabeled data. In (26), Zhang et al. proposed a novel uncertainty-guided mutual consistency learning framework for semi-supervised medical image segmentation. The model employs a dual-task backbone network with two output branches to simultaneously generate segmentation probability maps and signed distance maps. It performs intra-task consistency learning within self-ensemble tasks and utilizes task-level regularization for cross-task consistency learning to leverage geometric shape information. By estimating model segmentation uncertainty guidance, the framework effectively utilizes more reliable information from unlabeled data by selecting relatively determinis.

### 2.3 Pseudo-labeling

Pseudo-labeling involves generating targets for unlabeled data to obtain amplified, approximately fully labeled datasets (27). Common pseudo-labeling methods focus on effective pseudo-label generation strategies and how to generate high-quality segmentation results under the supervision of pseudo-labels. Wu et al. (28) proposed a novel Mutual Consistency Network (MC-Net) for semi-supervised left atrium segmentation in 3D MR images. MC-Net consists of an encoder and two slightly different decoders. It converts the prediction differences between the two decoders into unsupervised loss through a cyclic pseudo-labeling scheme to encourage mutual consistency. Building upon (28), Wu et al. (29) further introduced a model comprising a shared encoder and multiple slightly different decoders. This model represents the model’s uncertainty by computing the statistical differences among the outputs of multiple decoders, indicating uncertain regions in unlabeled data. The model obtains soft pseudo-labels using a sharpening function and applies a novel mutual consistency constraint between the probability output of one decoder and the soft pseudo-labels of other decoders. Chaitanya et al. (30) proposed a joint training framework defining per-pixel contrastive loss on pseudo-labels of unlabeled and sparsely labeled images, while applying traditional segmentation loss only on the labeled set. This method performs pseudo-label-based self-training and trains the network by jointly optimizing the contrastive loss proposed on labeled and unlabeled sets and the segmentation loss on the sparsely labeled set. Chen et al. (31) proposed a semi-supervised tissue segmentation framework called FDCT. This framework introduces the SBOM boundary refinement strategy, utilizing the characteristics of distance maps to optimize the pseudo-labels generated by the model, making them closer to the ground truth labels.

## 3 Proposed method based on multi-consistency learning

### 3.1 Overall structure

The task of semi-supervised segmentation of microscopic hyperspectral pathology images aims to learn more sample information by utilizing a small set of labeled samples and a large set of unlabeled samples. In this paper, we work with a dataset, denoted as D , which contains M labeled samples and N unlabeled samples, where M≪N We define the labeled dataset as $D^{L}={\left\{X^{i},Y^{i}\right\}}_{i-1}^{M}$ and the unlabeled dataset as $D^{U}={\left\{X^{i}\right\}}_{i=M+1}^{M+N}$ . Each sample $X^{i}\in {\mathbb{R}}^{H\times W\times C}$ in D is a microscopic hyperspectral image with a size of H×W and C channels. Correspondingly, $Y^{i}\in {\mathbb{R}}^{H\times W}$ is the segmentation label map associated with $X^{i}$ . The objective of semi-supervised segmentation is to learn a segmentation model $f_{s}\left(\theta_{s}\right)$ parameterized by $D=D^{L}+D^{U}$ from $\theta_{s}$ , such that each pixel in the input image is mapped to its correct class.

**Figure 2:**
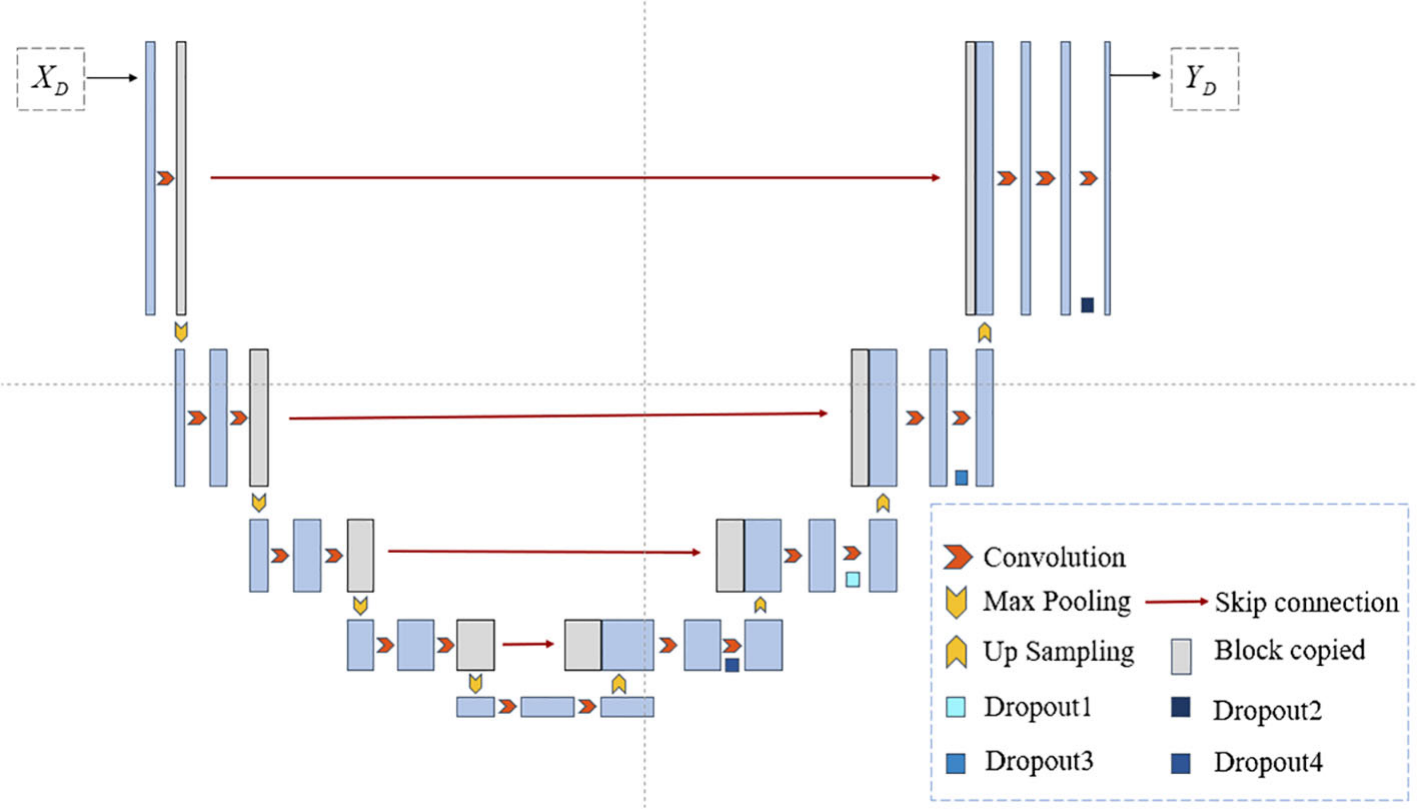
The U-Net structure used in this article.

The network model proposed in this paper is illustrated in **Figure 1**. During the training phase, in the preprocessing step on the left, Principal Component Analysis (PCA) is employed to perform dimensionality reduction on the input microscopic hyperspectral images. PCA serves a dual purpose: firstly, it helps distance the image from noise, thereby enhancing the data quality; secondly, it eliminates redundant spectral bands, reducing computational overhead and improving processing efficiency. We will use the data obtained by PCA dimensionality reduction as model input. In the segmentation step depicted in **Figure 1**, the feature vector FA is obtained by first passing through a shared encoder. Subsequently, various perturbations are applied to the feature vector FA, and the perturbed feature vectors are fed into different decoders. The outputs of multiple decoders are subjected to the proposed multi-consistency loss in this paper. All decoders update their model parameters during the training process. However, during testing, only one decoder is selected as the primary decoder, while the others are referred to as auxiliary decoders. Further details will be elucidated in Section 3.2. The U-Net model, known for its simple yet efficient structure, has found wide application in the field of medical image segmentation. Therefore, in our segmentation model, we employ an encoder-decoder structure based on U-Net, as depicted in **Figure 2**.

### 3.2 Training with multiple consistencies

After the input data D passes through the encoder E , it yields the feature vector FA. FA is directly fed into the primary decoder $D^{L}={\left\{X^{i},Y^{i}\right\}}_{i-1}^{M}$ . Different noise is introduced to the inputs of the model’s auxiliary decoders, represented as $FA_{i}=featurenoise\left(FA\right)$ , where *n*≥*i*> denotes the number of decoders and featurenoise() signifies the distinct noise added to FA. Specifically, we set n to 3 to strike a balance between effectiveness and training efficiency. Through experiments in Section 5.3, we ultimately determine featurenoise() to be Gaussian noise. Further experimental details will be presented in Sections 5.3 and 5.4.

The feature vector is processed by different decoders to obtain distinct probability maps. For the labeled dataset $D^{L}={\left\{X^{i},Y^{i}\right\}}_{i-1}^{M}$ this paper only calculates the supervised loss between the probability map from the primary decoder and its corresponding ground truth label. In this paper, the supervised loss is computed using both cross-entropy loss and Dice loss, defined by Equation (1):


(1) $$L_{s}=L_{CE}\left(y_{seg}^{L},Y\right)+L_{dice}\left(y_{seg}^{L},Y\right)$$

For the unlabeled data, we utilize the Soft-Hard process proposed in this paper to obtain pseudo-labels corresponding to different decoders for the same input. Firstly, we employ a sharpening function (32) to transform the probability maps into soft pseudo-labels. The computation process of the sharpening function is as Equation (2):


(2) $$PS=\frac{p{\left(y_{pred}|x;\theta \right)}^{1/T}}{p{\left(y_{pred}|x;\theta \right)}^{1/T}+{\left(1-p\left(y_{pred}|x;\theta \right)\right)}^{1/T}}$$

Here, T is a hyperparameter controlling the sharpening temperature. By choosing an appropriate T , we can apply entropy minimization constraint to regularize our model without introducing additional noise that might interfere with model training. The specific value of the temperature coefficient T will be tested in Section 5.5. Afterwards, we obtain the corresponding hard labels generated by the above process, denoted as PH=argmax(PS) . The process of obtaining hard labels can be expressed as Equation (3):


(3) $$PH=\arg\max\left(\frac{p{\left(y_{pred}|x;\theta \right)}^{1/T}}{p{\left(y_{pred}|x;\theta \right)}^{1/T}+{\left(1-p\left(y_{pred}|x;\theta \right)\right)}^{1/T}}\right)$$

By minimizing the consistency loss imposed on the update direction of the aforementioned constrained model, we enable the primary decoder to integrate sample features extracted by different decoders, thereby maximizing the learning of latent sample information from unlabeled samples. Ultimately, the overall loss function of this study is formulated as Equation (4):


(4) $$L_{u}=\sum_{i,j=1\&i\neq j}^{n}\left(L_{CE}\left(y_{seg}^{i},PH_{j}\right)+L_{dice}\left(y_{seg}^{i},PH_{j}\right)\right)$$

We treat the hard labels obtained from each decoder as the ground truth labels for the inputs of the other decoders. We then compute the multi-consistency loss between different decoders. Inspired by the findings in (32), we have adopted a strategy of weighting the supervised and unsupervised losses using hyperparameters to achieve improved experimental results. The formula for the multi-consistency loss is as Equation (5):


(5) $$L=\lambda L_{s}+\beta L_{u}$$

where λ represents the weight of the supervised loss, and β represents the weight of the unsupervised loss. Due to the lack of true labels for unlabeled data, pseudo-labels generated by the Soft-Hard process might initially lead the model in the wrong direction during training. Therefore, this paper adopts the method from (33) by adding a time-varying Gaussian weighting function, denoted as $\beta =0.001\times \exp\left(-5\times {\left(1-\frac{t}{t_{max}}\right)}^{2}\right)$ , to the unsupervised loss. This aims to balance the supervised loss and the consistency loss. Here, t denotes the current iteration count, and $t_{\max}$ represents the maximum number of iterations.

## 4 Experimental setup

### 4.1 Dataset

**Figure 3:**
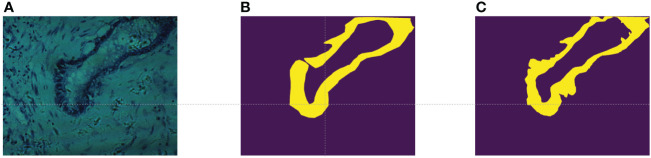
**(A)** False color images synthesized using bands 5,15, and 25; **(B)** The original label provided in (34); **(C)** Our re-labeling.

In this study, we utilized the cholangiocarcinoma micro-hyperspectral images from the multi-dimensional biliary tract database collected in (34). This dataset comprises 880 scenes from 174 individuals, with 689 scenes containing partially cancerous regions, 49 scenes representing complete cancerous regions, and 142 scenes devoid of any cancerous regions. The spatial resolution of these images is 1024×1280 pixels, each image encompassing 60 bands uniformly distributed from 550nm to 1000nm. While the database provides pixel-level labels for each image, it was observed through experimentation that these labels were somewhat coarse, failing to meet the accuracy requirements for semantic segmentation tasks. As illustrated in **Figure 3A**, shows a false-color image synthesized using the 5th, 15th, and 25th bands of the input data, while **Figure 3B** depicts the original labels provided by the database. It can be noted that, on one hand, the labels are disconnected at the opening of the circular structure, while in reality, they should be continuous. On the other hand, the boundaries between the tumor region and the normal region in the original labels are too sharp and abrupt, failing to accurately represent the true boundary information. Therefore, experienced researchers re-annotated the dataset, resulting in a total of 94 re-annotated images. The annotated results are shown in **Figure 3C**, demonstrating improved continuity and accuracy compared to the original labels.

### 4.2 Evaluation metrics

We employed four evaluation metrics to assess the performance of semi-supervised segmentation on histopathological images, including overall accuracy (OA), average accuracy (AA), Dice coefficient, and mean intersection over Union (MIoU). Better segmentation performance is indicated by higher values of OA, AA, Dice, and MIoU.

OA and AA represent the proportion of pixels correctly classified: We define the tumor region as positive samples and the non-cancer region (i.e., normal region) as negative samples. TP, TN, FP, and FN denote true positive pixels, true negative pixels, false positive pixels, and false negative pixels, respectively. Then, OA and AA can be defined as Equations (6) and (7):


(6) $$OA=\frac{TP+TN}{TP+TN+FP+FN}$$

(7) $$AA=\frac{1}{2}\left(\frac{TP}{TP+FN}+\frac{TN}{FP+TN}\right)$$

MIoU measures the ratio of the intersection to the union between the sets of true positive and predicted positive pixels as Equation (8). It provides a measure of how well the predicted segmentation aligns with the ground truth segmentation.


(8) $$MIoU=\frac{1}{2}\left(\frac{TP}{TP+FP+FN}+\frac{TN}{FP+FN+TN}\right)$$

The Dice coefficient is a metric that quantifies the similarity between the predicted segmentation (P) and the ground truth labels (Y) based on region overlap as Equation (9). It’s widely used in image segmentation tasks to evaluate the accuracy of the segmentation results.


(9) $$Dice=\frac{2\cdot \left|P\cap Y\right|}{\left|\text{P}\right|+\left|\text{Y}\right|}$$

### 4.3 Implementation detail

In this study, we implemented the model in the environment of PyTorch 1.13.1 with CUDA 11.7 and Python 3.8. Training and testing were performed on an NVIDIA GeForce RTX 3090. The batch size was set to 2, with batch sizes of 1 for labeled and unlabeled data, respectively. In the data preprocessing stage, the input data was dimensionally reduced to 6 channels using PCA. We utilized the SGD optimizer for training the entire network for 100 epochs, with a learning rate of 0.01 and momentum set to 0.9. Gaussian noise with mean 1 and standard deviation 1.2 was added to one auxiliary decoder, and mean 1 and standard deviation 1.5 was added to the other auxiliary decoder.

## 5 Result

### 5.1 Comparative experiments

To demonstrate the effectiveness of our proposed semi-supervised method, we conducted comparative experiments on the Multidimensional Cholangiocarcinoma Dataset. We compared our method with six other approaches, including:(1). 2D U-Net (35); (2) Mean Teacher (MT) (23); (3) Uncertainty Aware Mean Teacher (UA-MT) (33); (4) Cross Consistency Training (CCT) (36); (5) Cross Pseudo Supervision (CPS) (14); (6). Uncertainty-aware Pseudo-label and Consistency (UPC) (37); Here, the 2D U-Net is trained in a fully supervised manner using a limited set of labeled samples. Our proposed method and the other five approaches utilize semi-supervised learning algorithms with a certain proportion of labeled data and a large amount of unlabeled data.

**Figure 4:**
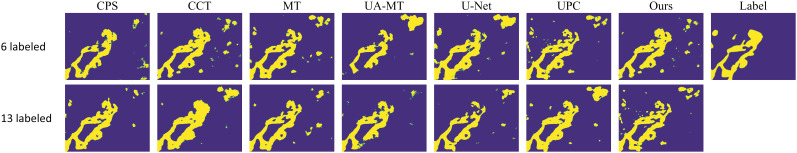
Visualizations of different methods.

**Table 1** presents quantitative results obtained using various semi-supervised models with different labeling ratios. From **Table 1**, it can be observed that our proposed method outperforms the fully supervised approach in scenarios with different labeling ratios. Particularly, when using 20% labeled data, our method shows improvements of 1.23% in OA, 0.77% in AA, 2.04% in MIOU, and 1.97% in DICE compared to the fully supervised 2D U-Net. This indicates that our proposed method is able to better utilize the information embedded in the unlabeled data compared to the fully supervised approach. In comparison with other semi-supervised methods, our method achieves results close to the other methods, indicating that our proposed approach is suitable for histopathological microscopic hyperspectral images. **Figure 4** displays the predicted results of the fully supervised and semi-supervised methods, including our proposed method, using different labeling ratios. It can be observed from the figure that our method’s predicted results are closer to the ground truth labels.

**Table 1:** Quantitative experimental results of different methods on multi-dimensional common bile duct dataset.

Labeled data	Method	OA ↑	AA ↑	Dice ↑	MIoU ↑
6/66(10%)	MT (23)	88.69%	82.34%	66.00%	69.24%
	UA-MT (33)	88.62%	82.93%	66.59%	69.35%
	CCT (36)	87.83%	82.69%	65.24%	68.21%
	CPS (14)	88.72%	82.83%	66.38%	69.53%
	UPC (37)	88.50%	83.32%	66.81%	69.65%
	2D-Unet (35)	88.56%	82.20%	65.24%	68.77%
	Ours	88.81%	82.76%	65.50%	68.95%
13/66(20%)	MT (23)	89.84%	**86.50%**	70.61%	72.45%
	UA-MT (33)	90.17%	86.05%	70.77%	72.67%
	CCT (36)	89.74%	85.96%	71.05%	72.79%
	CPS (14)	90.19%	86.03%	70.88%	72.59%
	UPC (37)	90.32%	85.11%	70.40%	72.53%
	2D-Unet (35)	89.25%	85.14%	69.15%	71.00%
	Ours	**90.48%**	85.91%	**71.19%**	**72.97%**

Bold text represent the optimal result.

The symbol '↑' signifies that a higher metric corresponds to better segmentation performance.

### 5.2 Ablation study

In order to validate the effectiveness of the proposed method, ablation experiments were conducted on the multi-dimensional bile duct dataset. We removed the “multi-consistency” learning method and the Soft-Hard pseudo-label generation method, constructing a “basic” model that uses a shared encoder and three independent decoders. We then separately added the multi-consistency learning strategy and the Soft-Hard pseudo-label generation method, referred to as “basic+mcl” and “basic+s-h” respectively. Next, we incorporated both of these methods into the “basic” model to obtain our final model, MCL-Net. The quantitative analysis results of the ablation study are presented in **Table 2**.

**Table 2:** Ablation results on multidimensional common bile duct dataset.

Labeled data	Method	Dice ↑	MIoU ↑
6/66(10%)	basic	69.43%	71.12%
	basic+mcl	70.50%	72.23%
	basic+s-h	70.69%	72.01%
	ours	71.19%	72.97%

The symbol '↑' signifies that a higher metric corresponds to better segmentation performance.

From **Table 2**, it can be observed that when using the “basic” model, the experimental results showed little improvement compared to the fully supervised approach. This indicates that merely employing a multi-decoder structure does not significantly enhance experimental performance. When we added our proposed multi-consistency learning strategy to the original model, the Dice coefficient and MIoU improved by 1.07% and 1.11% respectively. This demonstrates that the multi-consistency learning strategy effectively integrates features extracted by different decoders, extracting more sample information from unlabeled data.

When we separately added the Soft-Hard method to the model, the two evaluation metrics improved from 69.43% and 71.12% to 70.69% and 72.23%. This suggests that our proposed pseudo-label generation strategy can bring pseudo-labels closer to real labels to a greater extent. When we applied both of our proposed methods together on the model, experimental performance further improved. This indicates that our multi-consistency method and Soft-Hard method can collaborate synergistically to enhance experimental performance.

### 5.3 Influence of different data perturbation methods

To increase the diversity of input data among different decoders, we applied perturbations to the inputs of the two decoders, excluding the main decoder. We utilized three perturbation methods: adding Gaussian noise (Add-gn), adding salt-and-pepper noise (Add-spn), and adding Poisson noise (Add-pn). In our experiments, we employed strategies of using the same noise and adding different noises to different decoder inputs. Specifically, we denoted the use of Gaussian noise and salt-and-pepper noise as (Add-gspn), the use of Gaussian noise and Poisson noise as (Add-gpn), and the use of salt-and-pepper noise and Poisson noise as (Add-sppn). **Table 3** records the quantitative results corresponding to the six methods mentioned above. For each noise adding method, we conducted numerous experiments, and the results in **Table 3** represent the optimal settings for each method. From **Table 3**, it can be observed that the optimal experimental results are achieved when adding Gaussian noise to both auxiliary decoders. This may be attributed to the fact that Gaussian noise better simulates the existing noise patterns in images, aligning more closely with real-world requirements.

**Table 3:** Influence of different data perturbation methods.

Labeled data	Method	OA ↑	AA ↑	Dice ↑	MIoU ↑
6/66(10%)	**Add-gn**	**90.48%**	**85.91%**	**71.19%**	**72.97%**
	Add-spn	89.60%	84.28%	69.24%	71.51%
	Add-pn	88.77%	84.07%	70.61%	71.94%
	Add-gpn	90.45%	84.50%	70.81%	72.85%
	Add-gspn	89.59%	84.36%	69.29%	71.54%
	Add-sppn	89.72%	84.85%	69.23%	71.40%

Bold text represent the optimal result.

The symbol '↑' signifies that a higher metric corresponds to better segmentation performance.

### 5.4 Impact of different decoder numbers

In order to obtain more comprehensive sample features, we designed a multi-decoder structure. To better understand the influence of the number of decoders n on the experimental results, we set n to different values. When n=1 , the model corresponds to the classic 2D U-Net model. Since it is not possible to apply the proposed multi-consistency loss and Soft-Hard method in this case, the model operates in a fully supervised manner. When n≥1 , as described in section 5.3, experiments were conducted by perturbing the input data to the decoders using Gaussian noise applied before the auxiliary decoders.

**Figure 5:**
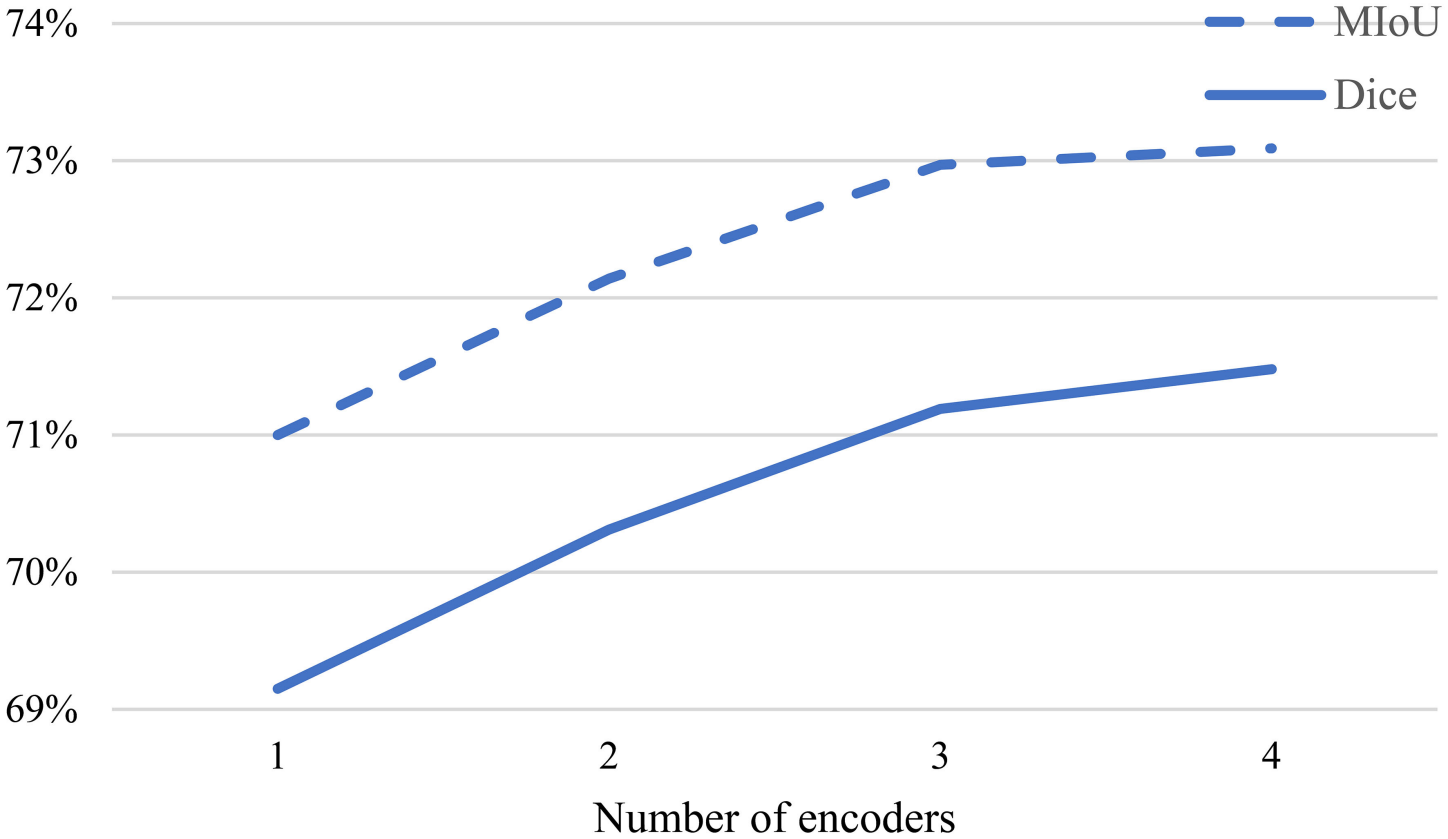
Impact of different decoder numbers on experimental results.

**Figure 5** illustrates the influence of the number of decoders on the Dice similarity coefficient under different proportions of labeled data. It can be observed from the figure that as n increases from 1 to 2, the Dice coefficient significantly improves. This indicates that the multi-decoder structure and multi-consistency learning strategy can comprehensively learn the model features. As n further increases from 2 to 3, the Dice coefficient continues to improve, suggesting that appropriately increasing the number of decoders allows for more effective utilization of information from unlabeled data. However, when n is increased to 4, the Dice coefficient experiences only a slight increase. Therefore, to balance accuracy and efficiency in the experiments, we set n=3 .

### 5.5 Impact of temperature T

**Figure 6:**
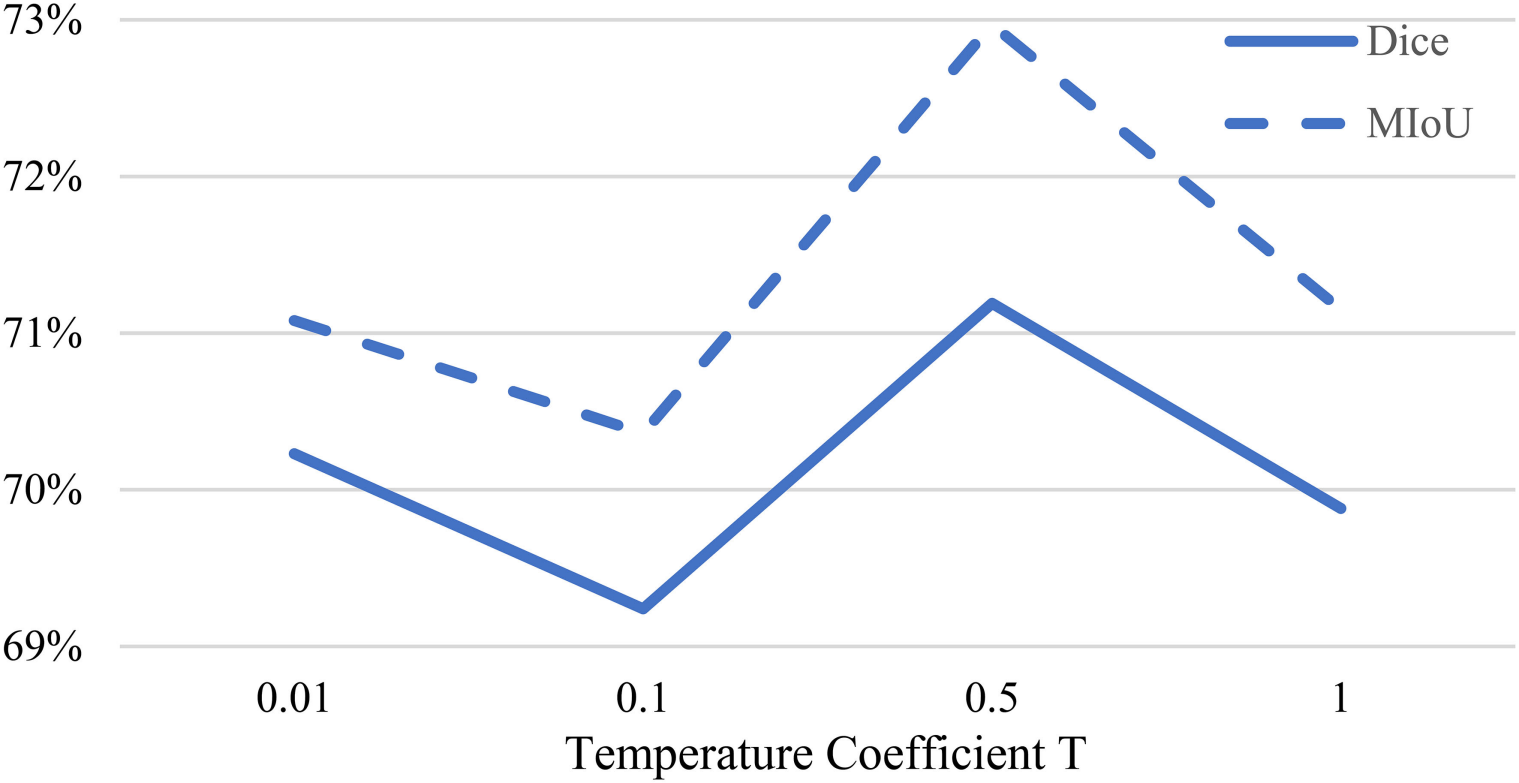
Impact of temperature coefficient T on experimental results.

In Equation 2, we use a sharpening function to generate preliminary pseudo-labels. **Figure 6** presents the Dice coefficients obtained by training our MCL-Net+ model on the multi-dimensional bile duct dataset with different temperature values T . Following the guidance from (29), we experimented with different values of T , specifically setting T to 0.01, 0.1, 0.5, and 1.

From **Figure 6**, it can be observed that when   , the model achieves the optimal experimental results. When T is too large, the model may fail to generate reasonable soft pseudo-labels due to the inability to utilize entropy minimization. On the other hand, when T is too small, it may introduce noise into the pseudo-labels, leading to prediction errors. Therefore, this study ultimately selects a temperature coefficient of T=0.5 .

### 5.6 Impact of supervised loss weight λ

**Figure 7:**
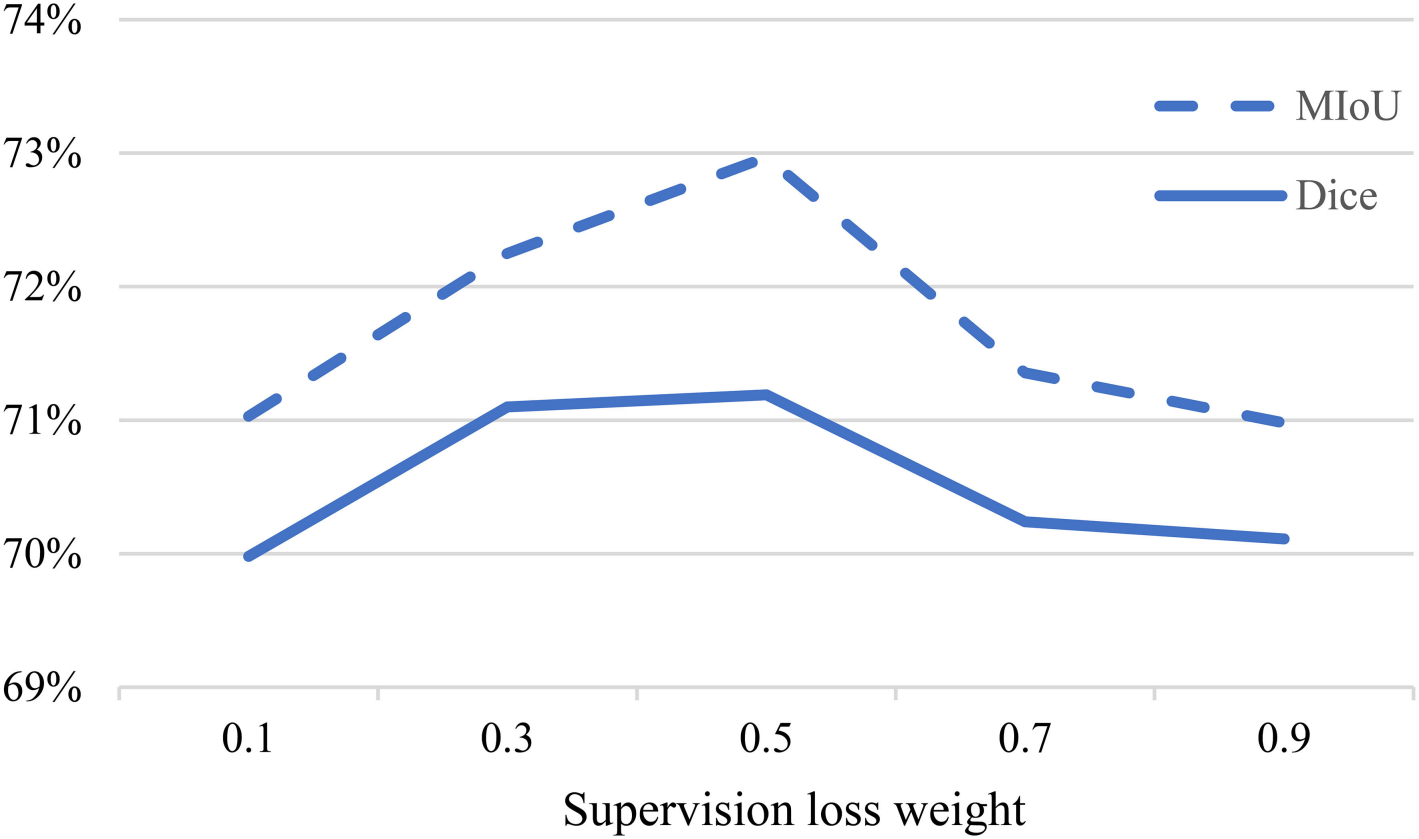
Impact of supervision loss weight on experimental results.

We further investigated the influence of the weight of the supervised loss term λ in the loss function. In Equation 5, the weight of the unsupervised loss is set according to a Gaussian warming function, while λ affects the balance between the two types of losses. **Figure 7** illustrates how different weights impact the experimental performance.

When λ is too high, the model tends to focus more on extracting features from the labeled data, but this comes at the cost of neglecting the unlabeled data, as the proposed multi-consistency loss may not be effectively utilized. On the other hand, when λ is too low, the accurately labeled data is not effectively leveraged, resulting in poorer experimental results. Therefore, we finally chose λ=0.5 as the final experimental setting.

## 6 Conclusion and future work

In this paper, we proposed a novel semi-supervised segmentation model, MCL-Net, for microspectroscopic pathology images. The model combines consistency regularization and pseudo-labeling methods. MCL-Net employs a shared encoder and multiple independent decoders. Through the proposed Soft-Hard pseudo-labeling strategy, MCL-Net generates pseudo-labels that are closer to the real labels for pathological images. Additionally, we introduced a multi-consistency learning strategy, treating the pseudo-labels generated by the Soft-Hard process as real labels. This encourages consistency among predictions from different decoders, enabling the model to learn more sample features.

The effectiveness of this approach was demonstrated through extensive experiments, providing a new perspective for the segmentation of microspectroscopic pathological images. Despite the promising results, there are limitations. Specifically, when using only 10% labeled data for experiments, our method did not significantly improve performance. This might be attributed to the limited explicit application of spectral information, which is unique to microspectroscopy. In the future, we will further explore ways to utilize spectral information and consider both labeled and unlabeled samples from multiple angles.

## Data availability statement

The datasets presented in this article are not readily available because this is for the use of this team only. Requests to access the datasets should be directed to JF, 2106020108@hhu.edu.cn.

## Ethics statement

Ethical approval was not required for the study involving humans in accordance with the local legislation and institutional requirements. Written informed consent to participate in this study was not required from the participants or the participants' legal guardians/next of kin in accordance with the national legislation and the institutional requirements.

## Author contributions

JF: Data curation, Formal Analysis, Investigation, Visualization, Writing – original draft, Writing – review & editing.
